# Efficacy of a Probiotic and Herbal Supplement in Models of Lung Inflammation

**DOI:** 10.3390/microorganisms10112136

**Published:** 2022-10-28

**Authors:** Nancy M. Wenger, Luhua Qiao, Teodora Nicola, Zoha Nizami, Xin Xu, Kent A. Willis, Namasivayam Ambalavanan, Amit Gaggar, Charitharth Vivek Lal

**Affiliations:** 1 ResBiotic Nutrition Inc., Birmingham, AL 35203, USA; nancy@resbiotic.com; 2 Division of Neonatology, Department of Pediatrics, University of Alabama at Birmingham, Birmingham, AL 35233, USA; luhuaqiao@uabmc.edu (L.Q.); tenicola@uabmc.edu (T.N.); drzohanizami@gmail.com (Z.N.); kentwillis@uabmc.edu (K.A.W.); nambalav@uabmc.edu (N.A.); 3 Division of Pulmonary and Critical Care Medicine, Department of Medicine, University of Alabama at Birmingham, Birmingham, AL 35233, USA; xinxu@uabmc.edu (X.X.); agaggar@uabmc.edu (A.G.); 4 Program in Protease and Matrix Biology, University of Alabama at Birmingham, Birmingham, AL 35233, USA

**Keywords:** lung inflammation, probiotic supplement, chronic pulmonary disease

## Abstract

Background: Gut microbiome dysbiosis is associated with lung disease through the gut-lung axis. Abundant proteobacteria increase MMP-9 and contribute to tissue proteolysis followed by neutrophil recruitment, lung tissue injury, and perpetuation of chronic lung disease. We sought to determine if a scientifically formulated probiotic and herbal supplement could attenuate neutrophilic inflammation and improve lung structure and function in models of lung inflammation. Methods: For in vitro experiments, epithelial cells exposed to proteobacteria were treated with resB—a blend of three probiotic Lactobacillus strains and turmeric, holy basil, and vasaka herbal extracts. For in vivo experimentation, mice exposed to pulmonary proteobacteria-derived lipopolysaccharide were treated by gavage with resB. Results: In vitro, the bacterial and herbal components of resB decreased activity of the MMP-9 pathway. Mice exposed to LPS and pre- and post-treated with resB had decreased neutrophil recruitment and inflammatory biomarkers in bronchoalveolar lavage fluid, serum, and lung tissue compared to untreated mice. Conclusions: This study describes the mechanisms and efficacy of probiotic and herbal blend in pre-clinical models of lung injury and inflammation.

## 1. Introduction

Chronic pulmonary diseases make up a significant portion of global burden of disease, with chronic obstructive pulmonary disease accounting for 3.2 million deaths and asthma accounting for 495,000 deaths annually [1]. With emergent factors such as air pollution and the COVID-19 pandemic threatening respiratory health, supporting lung health has become globally important.

Microbial imbalances in the microbiome have been associated with chronic lung diseases [2,3,4,5,6]. As our group and others have previously shown, increased proteobacteria in the lungs can upregulate the activity of matrix metalloproteinase 9 (MMP-9), leading to progressive lung tissue damage and the development of chronic lung diseases [7,8,9,10,11,12,13,14,15]. This cascade results in collagen degradation generating tripeptide N-acetyl proline-glycine-proline (Ac-PGP), a potent neutrophil chemoattractant [9,10,16,17,18]. Neutrophilic inflammation is a common feature of many chronic lung diseases, and other cytokines including C-reactive protein (CRP) may contribute to neutrophil recruitment and increased inflammatory response [19,20,21].

Intestinal microbiota and nutrition can have a systemic impact on the health of the lungs; this effect is known as the gut-lung axis. A promising route to ameliorating inflammation in the lungs and supporting healthy respiratory function may be through the action of the gut-lung axis. Gut bacteria enter the respiratory tract directly through constant microaspiration [22]. In addition, nutrients consumed orally are broken down and converted by gut bacteria to metabolites that travel through systemic circulation and modulate immune and inflammatory responses [23]. Dysbiosis in the gut can trigger pro-inflammatory pathways and be exacerbated with poor nutrient intake [24,25]. Chronic lung diseases are often associated with dysbiosis-related gastrointestinal symptoms [5,26]. Therefore, nutrition and balanced gut microbiota are key components of a multi-factorial approach to support proper lung function [22,25,27].

Live bacterial strains of the genus *Lactobacillus* have been widely studied in humans for oral supplementation. Oral administration of *Lactobacillus plantarum* has shown efficacy in improving upper respiratory tract infections and *Lactobacillus rhamnosus* in reducing risk of respiratory and gastrointestinal infections [28,29]. *Lactobacillus acidophilus* mediates anti-inflammatory short chain fatty acid (SCFA) uptake in the gut epithelium [30]. Furthermore, the consumption of herbal extracts of holy basil leaf, turmeric root, and vasaka leaf shows potential in supporting bronchodilatory, antioxidant, and anti-inflammatory function through the gut-lung axis [31,32,33]. In this study, we hypothesized that a probiotic with antioxidant herbal extracts could attenuate neutrophilic inflammation in vitro and in animal models.

## 2. Materials and Methods

### 2.1. resB Composition

Probiotic blend- resB blend #1

- Lactiplantibacillus plantarum RSB11^TM^
- Lactobacillus acidophilus RSB12^TM^
- Lacticaseibacillus rhamnosus RSB13^TM^

Turmeric—*Curcuma longa*, root (EC330227, Naturex)

Holy basil—*Ocimum sanctum*, leaves (50287, Star Hi Herbs)

Vasaka—*Adhatoda vasica*, leaves (VAS, Karallief)

### 2.2. In Vitro Model

Human bronchial epithelial cells (HBECs) (American Type Culture Collection, Manassas, VA, USA; product no. PCS-300-010) or human intestinal epithelial cells (IECs) (American Type Culture Collection, product no. HTB-37) were seeded on 6-well plates at 4 × 10^5^ cells density. Sixteen hours later, medium was changed to fresh antibiotic-free DMEM/F12.

*Escherichia coli* (ATCC 25922) was added to IECs at final concentrations of 5 × 10^7^ CFU/mL followed by a 5-fold or 2-fold serial dilution. Treatment with live probiotic strains in IECs started at 2.5 × 10^8^ CFU/mL followed by 10-fold serial dilutions of the strains. Similarly, for HBECs, E. coli exposure was performed with 5 × 10^7^ CFU/mL. Cells were treated with herbal extracts at 10 mg/mL initial concentration followed by 2-fold serial dilutions and/or a with a blend of equal parts of RSB11, RSB12, and RSB13 constituting resB blend #1 at a final concentration of 5 × 10^7^ CFU/mL. Cells were incubated at 37 °C for 4 h and RNA was extracted with TRIzol^TM^ lysis reagent (15596026; Invitrogen, Waltham, MA, USA).

### 2.3. Bacterial Growth Inhibition Assay

Each herbal extract of turmeric, holy basil, and vasaka was resuspended in PBS at 200 mg/mL initial concentration followed by 2-fold serial dilutions and PBS control. Bacteria strains RSB11, RSB12, and RSB13 were cultured individually overnight, mixed at a concentration of 1 × 10^7^ CFU/mL with herbs, and incubated at 37 °C for 4 h. The optical density of the strains was measured, and dilution factor calculated. MRS (de Man, Rogosa & Sharpe) agar plates were smeared in triplicate with the diluted bacterial samples and incubated for 16 h. Following the incubation period, colonies were counted on each plate.

### 2.4. LPS Inflammation Mouse Model

All animal protocols were submitted and approved by the Institutional Animal Care and Use Committee of UAB (IACUC-20828). All mouse experiments were carried out in accordance with National Institutes of Health guidelines. C57BL/6J male and female mice were used to generate a 4-day LPS (*P. aeruginosa*) model with resB treatment.

To generate a 4-day dysbiosis model, C57BL/6J mice (Jackson Laboratories, ME, USA) at 6-8 weeks of age were divided into three groups of 6 mice each (3M/3F):

1. resB + saline
2. Saline + LPS
3. resB + LPS

Mice were gavaged with resB or saline control on Day 1, dosed intratracheally (IT) with LPS on Day 2, and gavaged with resB or saline control on Days 3 and 4 before sacrifice on Day 5.

The representative resB formulation administered to mice contained 3 × 10^6^ CFU resB blend #1 (1 × 10^6^ each strain), 0.34 mg turmeric, 0.48 mg holy basil, and 0.56 mg vasaka per dose. Herb doses were determined based on effective human doses of 50 mg turmeric, 70 mg holy basil, and 80 mg vasaka converted to the appropriate mouse dose based on prior literature [34]/ On day 1, mice were gavaged with 150 μL saline or 150 μL resB using a feeding needle (FTP-20-30, 20GAx30 mm; Instech). On day 2, all mice excluding controls were dosed intratracheally (IT) with lipopolysaccharide (LPS) (*P. aeruginosa* ATCC27316; Sigma, St. Louis, MO, USA) at a concentration of 100 µg/50 µL PBS/mouse to generate lung injury. Controls were dosed IT with saline. On days 3 and 4, mice were gavaged with 150 μL saline or 150 μL resB.

*Tissue Harvest*- On day 5, mice were euthanized. Bronchoalveolar lavage (BAL) fluid was collected by injecting and removing 0.25 mL of PBS from the right lung via the trachea. The right lung was collected and frozen for lung tissue. Blood was collected by cardiac puncture and serum isolated by centrifuging the samples at 5000× *g* for 15 min at 4 °C. Samples were stored at −80 °C until analysis.

*BAL Cell Count*- BAL was spun down and the pellet resuspended in 200 μL PBS. Total cells were counted with a hemacytometer. For differential cell count, BAL cells were spun down onto slides with a cytospin centrifuge, stained using Hema 3^TM^ Stain as per manufacturer’s instructions (23-123869; Fisher Healthcare, Waltham, MA, USA), and then counted by type.

### 2.5. Measurement of BAL and Serum Myeloperoxidase, MMP-9, and CRP Protein Concentration

In all mice, MPO concentration in BAL was measured using the Mouse Myeloperoxidase DuoSet ELISA (DY3667; Biotechne, Minneapolis, MN, USA), MMP-9 concentration in BAL and serum using the Mouse Total MMP-9 DuoSet ELISA (DY6718; Biotechne, Minneapolis, MN, USA), and CRP protein in serum using the Mouse C-Reactive Protein/CRP DuoSet ELISA (DY1829; Biotechne, Minneapolis, MN, USA) per manufacturer’s instructions. After each target analyte was captured by its respective bead, a detection antibody was introduced. This was incubated with Streptavidin-PE conjugate completing the reaction on the microbead surface. Luminescence was measured by Luminex MAGPIX analyzer, calculating the average for each cytokine set of beads. Washings and incubations strictly followed the manufacturer’s protocols. Multiple conjugated beads facilitated obtaining multiple results for each sample. Data was normalized to a standard curve.

#### 2.5.1. Measurement of MMP-9 mRNA Expression

Total RNA from cells and homogenized lung tissue was extracted using TRIzol lysis reagent (15596026; Invitrogen, Waltham, MA, USA) and reverse transcribed using the SuperScript^®^ III First-Strand Synthesis System for RT-PCR (18080-051, Invitrogen) per manufacturer’s instructions. Quantitative real-time PCR (RT-PCR) was performed using primer-probes for human MMP-9 (Hs00957562_m1, Thermo Fisher) and mouse MMP-9 per manufacturer’s instructions (Mm00442991_m1, Thermo Fisher). RT-PCR was performed on the MyiQ™ Single-Color Real-Time PCR detection System (Bio-Rad, Hercules, CA, USA) using SYBR Green PCR Master Mix per manufacturer’s instructions (4309155; Applied Biosystems, Waltham, MA, USA). RT-PCR was performed using an initial 10 min denaturation period at 95 °C followed by 50 cycles of 15 s at 95 °C and 1 min annealing and extension at 60 °C. Expression levels of MMP-9 were normalized to 18S RNA.

#### 2.5.2. Statistical Analysis

Analysis was performed using one-way ANOVA with Tukey’s multiple comparison test. All data analysis was performed using GraphPad Prism version 9.2 (GraphPad Software, La Jolla, CA, USA). Data is reported as mean ± standard deviation, and representative of at least two independent experiments.

## 3. Results

### 3.1. E. coli Colonization Triggers Neutrophilic Inflammation in In Vitro Intestinal Cell Model of Bacterial Imbalance

**Figure 1:**
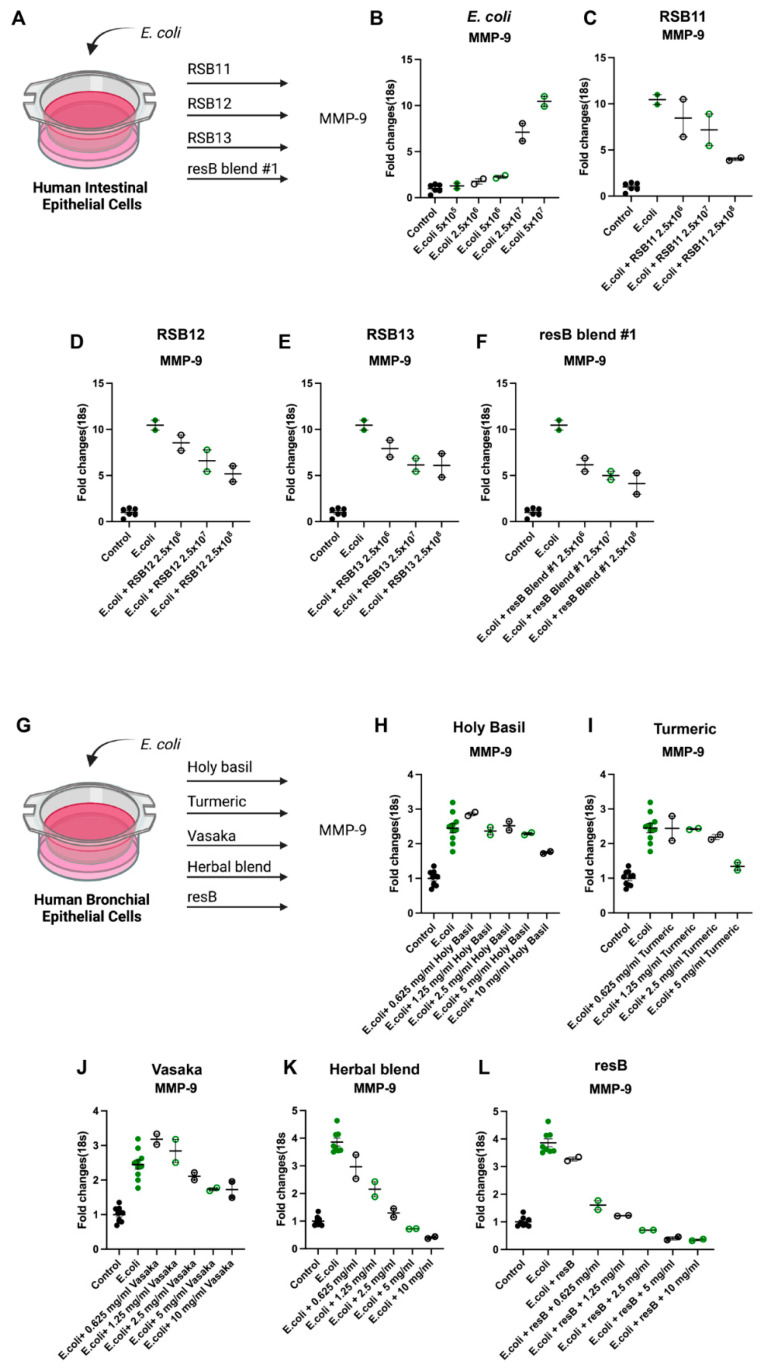
resB blend #1 (RSB11, RSB12, RSB13) and herbal extracts counteract neutrophilic inflammation via the MMP-9 pathway in human IEC and HBEC models of proteobacteria abundance in the gut and lung. (**A**) Cells were exposed to *E. coli* before treatment with probiotic strains. Schematic created in Biorender. (**B**) MMP-9 transcription increases upon addition of increasing concentrations of *E. coli* in a human intestinal cell line model of dysbiosis. (**C**) RSB11, (**D**) RSB12, (**E**) RSB13, and (**F**) resB blend #1 (RSB11, RSB12, RSB13) decrease MMP-9 in IECs exposed to *E. coli.* (**G**) Cells were exposed to *E. coli* before treatment with increasing dosages of (**H**) holy basil, (**I**) turmeric, (**J**) vasaka, (**K**) herbal extract blend, or (**L**) resB blend #1 probiotic blend (RSB11, RSB12, RSB13) with increasing dosages of herbal extract blend that each led to decreases in MMP-9.

To understand the relationship between proteobacteria abundance and neutrophilic inflammation in the gut microenvironment, we created an in vitro model of gut dysbiosis. IECs were exposed to *E. coli* at increasing concentrations (Figure 1A). We observed increased MMP-9 mRNA upon exposure to *E. coli* (Figure 1B) and reduced levels upon treatment with individual strains RSB11, RSB12, and RSB13 (Figure 1C–E) and these strains as a blend (resB blend #1) (Figure 1F).

### 3.2. Probiotics and Herbal Extracts Decrease MMP-9 Expression in In Vitro Model of Lung Bacterial Imbalance

To determine the anti-inflammatory activity on the respiratory epithelium, we treated an HBEC model of dysbiosis with herbal extracts and resB blend #1 (Figure 1G). Both the herbs alone (Figure 1H–K) and the herbs in combination with resB blend #1 (Figure 1L) resulted in decreased MMP-9 expression.

### 3.3. Herbal Extracts Do Not Inhibit Colony Growth of RSB Strains

**Figure 2:**
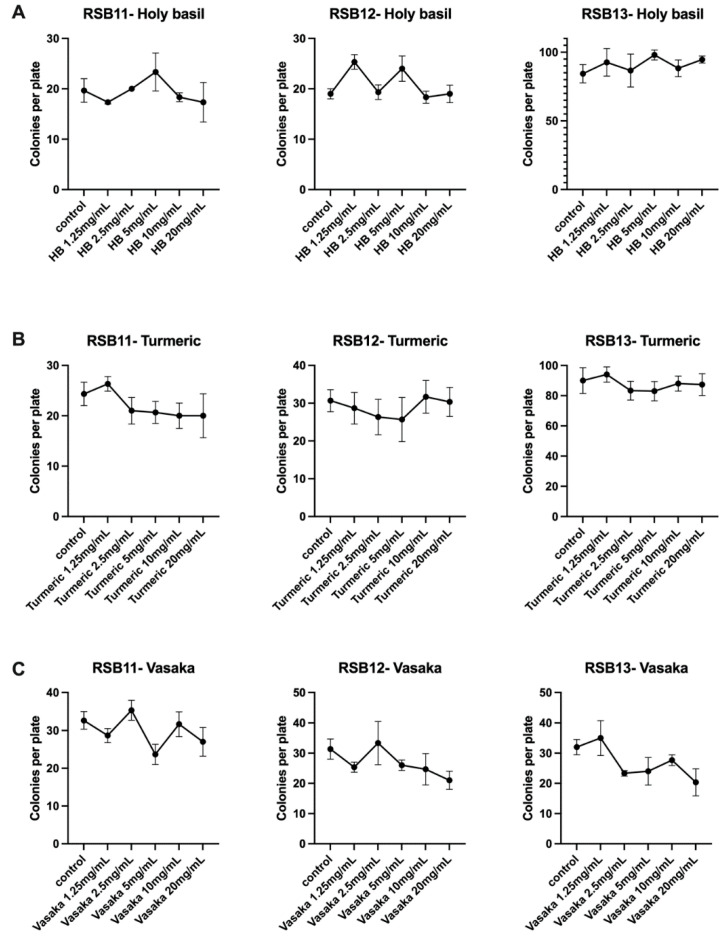
Holy basil, turmeric, and vasaka extracts do not inhibit colony growth of RSB11, RSB12, or RSB13 strains. (**A**) Holy basil extract. (**B**) Turmeric extract. (**C**) Vasaka extract. Holy basil, turmeric, and vasaka extracts do not inhibit colony growth of RSB11, RSB12, or RSB13 strains. (**A**) Holy basil extract. (**B**) Turmeric extract. (**C**) Vasaka extract.

To verify that the selected herbal extracts would not interfere with the survival of the live probiotic strains, we conducted an in vitro growth inhibition assay. Turmeric, holy basil, and vasaka extracts were added at increasing concentrations to individual RSB11, RSB12, and RSB13 strains. After overnight incubation, none of the three herbal extracts inhibited colony growth of any of the three probiotic strains (Figure 2).

### 3.4. Oral Delivery of Probiotic and Herbal Formulation Decreases Markers of Neutrophilic Inflammation in Murine Model of Respiratory Bacterial Imbalance and Injury

**Figure 3:**
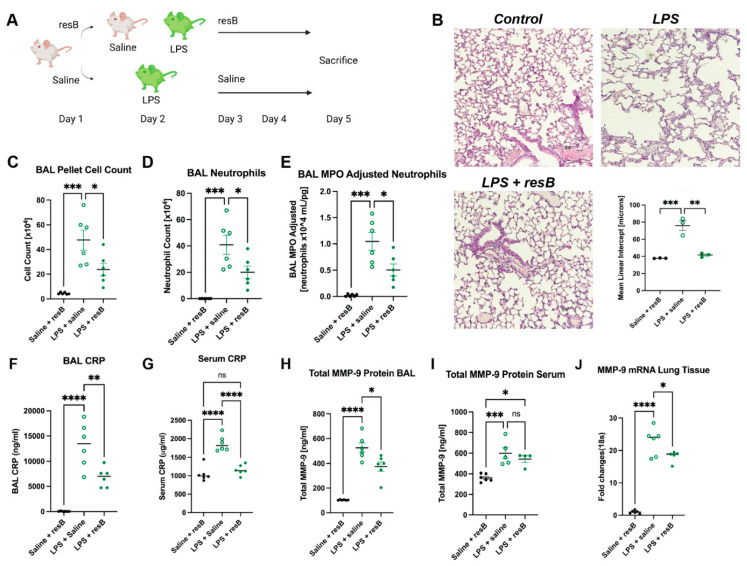
resB decreases neutrophilic inflammation in an in vivo model of proteobacterial dysbiosis. (**A**) Mice were exposed to LPS intratracheally before treatment with saline or resB. Schematic created in Biorender. (**B**) Representative histology sections of lungs show decreased mean linear intercept (MLI) in LPS + resB mice compared to mice exposed to LPS alone. 10× magnification, scale bar 100 μm. (**C**) BAL sample cell count decreases significantly in LPS + resB mice. (**D**) BAL neutrophil count decreases significantly in LPS + resB mice. (**E**) BAL MPO (adjusted for neutrophils 10^4^ mL/pg) decreases significantly in LPS + resB mice. (**F**) BAL CRP decreases significantly in LPS + resB mice. (**G**) Serum CRP decreases significantly in LPS + resB mice. (**H**) Total BAL MMP-9 protein decreases significantly in LPS + resB mice. (**I**) Total serum MMP-9 protein is not significantly decreased in LPS + resB-treated mice. (**J**) Lung tissue MMP-9 mRNA decreases significantly in LPS + resB mice. * *p* < 0.05, ** *p* < 0.01, *** *p* < 0.001, **** *p* < 0.0001 by one-way ANOVA with Tukey’s post hoc test.

We developed a murine model of proteobacteria-induced respiratory inflammation to investigate the anti-inflammatory impact of oral supplementation with resB, the formulation of probiotic resB blend #1 and herbal extracts (Figure 3A). Then, 6- to 8-week-old C57BL/6J mice were treated with a combination of resB + saline, LPS + saline, or LPS + resB. Mice were exposed to intratracheal LPS or saline (control) on Day 2 to generate lung inflammation and injury. Mice were treated with resB or saline (control) by gavage on Days 1, 3, and 4 before euthanasia on Day 5. No within-group differences were observed between male and female mice. Mice exposed to LPS showed a characteristic breakdown in lung tissue structure confirmed by MLI values that was reduced in severity by dosing with resB (Figure 3B).

In order to assess neutrophil influx in the lungs in response to dysbiosis, we conducted cell counts in BAL. Total cell count and neutrophil cell count in BAL increased in mice exposed to LPS compared to the control and decreased significantly when LPS-exposed mice were given resB (Figure 3C,D). Accordingly, myeloperoxidase (MPO) and CRP levels followed the same pattern—rising with LPS exposure and decreasing with resB treatment (Figure 3E–G). MMP-9 protein levels in BAL increased in the LPS exposure group and decreased in the resB group as expected (Figure 3H). However, serum MMP-9 protein showed no significant difference between the LPS and resB groups, although both showed elevated levels compared to the control (Figure 3I). In lung tissue, MMP-9 mRNA increased in the LPS exposure group and decreased when treated with resB (Figure 3J).

## 4. Discussion

In this study, we established in vitro and in vivo models of proteobacteria-driven inflammation and injury and assessed the mechanisms and safety of resB, a probiotic blend with herbal extracts. We showed that *E. coli* exposure in vitro was sufficient to induce MMP-9 activity in intestinal and respiratory epithelial cells. Probiotic *Lactobacillus* strains and herbal extracts individually and blended attenuated this induction of MMP-9.

We also studied the gut-lung axis action of resB in an in vivo model of proteobacteria-induced inflammation. Lung structure and inflammation improved significantly upon oral resB treatment compared to untreated mice exposed to pulmonary LPS alone. Neutrophilic inflammation was reduced in the lungs upon resB treatment based on decreases in BAL neutrophils, MPO and MMP-9 protein, and lung tissue MMP-9 mRNA. CRP is known to act as a part of the body’s first-line immune response and increases in an inflammatory response [35], and both serum and BAL CRP decreased with resB supplementation.

In mice given resB we observed a decrease of MMP-9 and CRP in lungs and a decrease in CRP, but not MMP-9, in the serum. Further analysis of human serum and mouse lung samples for known regulators of the MMP-9 pathway such as LTA4H may supplement our understanding of its modulation [11]. Additionally, investigation of the mechanisms of action of the herbal extracts is warranted in animal models, as MMP-9 is not the only pathway through which they reduce inflammation. The alkaloid fraction from vasaka shows anti-inflammatory effects through the TNF-α pathway, and curcumin isolated from turmeric may downregulate NF-κB, IL-1, and IL-6 [31,36].

Mouse models are useful for exploring mechanisms of action but are limited in accurately modeling the multi-factorial dynamics of a human microbiome. Future studies exploring the action of the gut-lung axis and the role of probiotics will be best examined in humans. Short chain fatty acids are a category of anti-inflammatory molecules whose production is increased upon probiotic administration and can be measured in serum and stool samples [37,38]. A 16s analysis of gut and lung microbiome changes in patients with respiratory conditions would also support the ability of oral supplementation to modulate the microbiome and inflammation via the gut-lung axis.

## 5. Conclusions

In summary, we have implicated the MMP-9 pathway in the relationship between proteobacteria-induced injury and resultant neutrophilic inflammation. Dosing a probiotic plus herbal supplement in in vitro and in vivo models of dysbiosis reduced neutrophilic inflammation in the lungs characteristic of many chronic pulmonary diseases. Future studies may build on this finding by exploring additional pathways at work in this inflammatory response. With increasing threats to lung health in today’s world, the possible clinical relevance of these findings may affect both patients with existing chronic pulmonary disease and patients hoping to protect their lung health against inflammatory stimuli. These findings may pave the way for a clinical study to determine safety of a probiotic and herbal supplement targeting the gut-lung axis.

## Data Availability

The data would be available as an open access publication. No publicly available data is available.
